# Intra-night variation in apnea-hypopnea index affects diagnostics and prognostics of obstructive sleep apnea

**DOI:** 10.1007/s11325-019-01885-5

**Published:** 2019-07-11

**Authors:** Sami Nikkonen, Juha Töyräs, Esa Mervaala, Sami Myllymaa, Philip Terrill, Timo Leppänen

**Affiliations:** 1 Department of Applied Physics, University of Eastern Finland, Kuopio, Finland; 2 Department of Clinical Neurophysiology, Diagnostic Imaging Center, Kuopio University Hospital, Kuopio, Finland; 3 School of Information Technology and Electrical Engineering, The University of Queensland, Brisbane, Australia; 4 Department of Clinical Neurophysiology, Institute of Clinical Medicine, Faculty of Health Sciences, University of Eastern Finland, Kuopio, Finland

**Keywords:** OSA, AHI, Diagnostics, Prognostics

## Abstract

**Background:**

Diagnostics of obstructive sleep apnea (OSA) is based on apnea-hypopnea index (AHI) determined as full-night average of occurred events. We investigate our hypothesis that intra-night variation in the frequency of obstructive events affects diagnostics and prognostics of OSA and should therefore be considered in clinical practice.

**Methods:**

Polygraphic recordings of 1989 patients (mean follow-up 18.3 years) with suspected OSA were analyzed. Number and severity of individual obstructive events were calculated hourly for the first 6 h of sleep. OSA severity was determined based on the full-night AHI and AHI for the 2 h when the obstructive event frequency was highest (AHI_2h_). Hazard ratios for all-cause, cardiovascular, and non-cardiovascular mortalities were calculated for different OSA severity categories based on the full-night AHI and AHI_2h_.

**Results:**

Frequency and duration of obstructive events varied hour-by-hour increasing towards morning. Using AHI_2h_ led to a statistically significant rearrangement of patients between the OSA severity categories. The use of AHI_2h_ for severity classification showed clearer relationship between the OSA severity and mortality than the full-night AHI.

**Conclusions:**

Currently, the intra-night variation in frequency and severity of obstructive events is completely ignored by conventional, full-night AHI and considering this information could improve the diagnostics of OSA.

## Introduction

The diagnostics of obstructive sleep apnea (OSA) is currently based on apnea-hypopnea index (AHI), which is simply the number of apnea and hypopnea events per hour of sleep [1]. Currently, OSA is diagnosed if AHI ≥ 5 and the patient experiences OSA-related symptoms such as daytime sleepiness. Alternatively, AHI ≥ 15 even without any symptoms is enough for diagnosis. However, the current diagnostics based on AHI has some shortcomings. For example, AHI is calculated as a full-night average and thus provides no information whether the frequency or severity of obstructive events changes during the night. In addition, the used hypopnea scoring rules have a large effect on AHI [2]. Furthermore, it has been reported multiple times that AHI may not be the best parameter for diagnosing OSA and estimating its severity [3–7]. Despite these shortcomings, diagnosis of OSA is currently based on AHI.

There are some previous studies regarding the change in OSA severity during the night [8–10]. However, the number of patients in these studies has been relatively small and obstructive event severity and frequency during the night have not been studied on hour-by-hour basis. Charbonneau et al. reviewed 66 patients with severe OSA and found that as the night continued, the mean apnea duration increased from 27.2 s in the first quartile of the recording to 34.6 s in the last quartile of the recording and AHI increased from 70.4 to 76.1 [8]. Furthermore, Oksenberg et al. found that OSA severity in severe OSA increases through the night even when the change in body posture is taken into account [10]. They concluded that body position did not significantly affect the change in OSA characteristics during the night [10]. Importantly, Gami et al. reported that in OSA patients, 46% of sudden deaths from cardiac causes occurred between midnight and 6 a.m. while in people without OSA, this percentage was 21% [9].

We hypothesize that the severity of the obstructive events increases towards the morning. Our hypothesis is based on the knowledge that later during the night, larger proportion of time is spent in rapid eye movement (REM) sleep and obstructive events during REM sleep are more severe [11, 12]. In light of the previous studies [8–10], we expect that AHI increases towards morning also in the present study population. In addition, we hypothesize that if only the hours with the highest number of obstructive events are used as a basis of OSA diagnosis, the estimation of OSA severity and the mortality risk caused by OSA can be improved.

To evaluate the validity of our hypotheses, we investigate, using a large patient cohort (*n* = 1989), how the frequency and severity of obstructive events change during the night on hour-to-hour basis. In addition, we study how this variation affects the patients’ prognosis with long follow-up time (mean 18.3 years) and whether these changes should be taken into account in diagnostics and prognostics of OSA.

## Methods

The study is based on a retrospective reanalysis of 1989 polygraphic recordings of patients (Table 1) with suspected OSA. The data was collected with a custom-made four-channel (airflow, respiratory effort, body position, blood oxygen saturation) ambulatory device during 1992–2003 in Kuopio University Hospital, Kuopio, Finland [13]. All recordings were reanalyzed according to scoring rules of American Academy of Sleep Medicine (AASM 2007) [14] and clinical practice of Kuopio University Hospital at the time of analysis. Apnea event was scored if the airflow signal dropped ≥ 90% from reference level for at least 10 s. Hypopnea event was scored if the airflow signal dropped ≥ 30% from reference level for ≥ 10 s causing at least 4% peripheral oxygen desaturation. Since the recordings used in this study did not include electroencephalography (EEG), arousals could not be detected and thus were not taken into account when scoring hypopneas. Obvious periods of activity were identified as wake and the remaining periods were presumed as sleep in the analyses. Patient information was collected from patients’ medical records in Kuopio University Hospital in 2013. Causes of death were obtained from Statistics Finland (Helsinki, Finland) in 2018. Ethics Committee of the Hospital District of Northern Savo, Kuopio, Finland, approved the collection and analysis of the polygraphic recordings (127/2004 and 24/2013).

**Table 1:** The patient demographic data: median (range) for continuous parameters and *N* (% of valid cases) for categorical variables in non-OSA, mild, moderate, and severe obstructive sleep apnea categories. The parameter “missing” represents the number of patients of which the information in question was not available

Severity classification	Non-OSA	Mild	Moderate	Severe
Number of patients	967	505	257	260
Follow-up time (years)	19.4 (0.2–26.0)	19.1 (0.0–26.0)*	17.8 (1.0–25.7)	17.8 (0.1–25.9)
Age (years)	45.8 (18.3–69.6)*	50.3 (25.4–79.9)	50.2 (27.8–80.3)	51.6 (20.6–81.1)
BMI (kg/m^2^)	26.6 (17.5–63.3)*	28.7 (18.7–60.6)*	30.7 (19.2–56.1)*	34.5 (21.1–74.0)
Missing (*N*)	60	12	2	3
AHI_standard_ (1/h)	1.5 (0.0–4.9)*	8.6 (5.0–14.9)*	20.8 (15.1–29.8)*	47.6 (30.0–148.7)
Male gender	643 (66.5)	392 (77.6)	213 (82.9)	232 (89.2)
Smoking				
Yes	274 (31.8)	136 (28.6)	60 (25.8)	79 (32.2)
Quit	198 (23.0)	128 (26.9)	74 (31.8)	84 (34.3)
No	389 (45.2)	212 (44.5)	99 (45.2)	82 (33.5)
Missing	106	20	24	15
CPAP	48 (5.0)#	96 (19.0)#	100 (38.9)#	148 (57.1)
Missing (*N*)	15	1	0	1

The patients were divided into the obstructive sleep apnea categories based on conventional full-night apnea-hypopnea index (AHI_standard_). An asterisk (*) and a hashtag (#) denote statistically significant (*p* < 0.05) difference between the marked category and one level more severe obstructive sleep apnea category. Statistical significances were evaluated using Mann-Whitney *U* test (*) and chi-square test (#)

In the first part of the study, only those patients that had AHI ≥ 5 and who were considered to sleep at least 6 h (*n* = 922) were included in the analysis. The patients were divided into the standard OSA severity categories, defined by AASM (i.e., mild 5 ≤ AHI < 15, moderate 15 ≤ AHI < 30, and severe AHI ≥ 30) [1]. The first 6 h of sleep for each patient were analyzed and parameters describing the severity of OSA and individual obstructive and desaturation events were calculated using custom MATLAB (MathWorks, Natick, MA) functions. The calculated parameters were AHI, average apnea duration, average hypopnea duration, oxygen desaturation index (ODI), average desaturation duration, and the apnea proportion of all obstructive events. The definitions of the parameters are presented in Table 2. Finally, hourly medians of all parameters were calculated for all patients in each OSA severity category and the change in the values across night was assessed in hour-by-hour basis.

**Table 2:** Definitions of the parameters used in this study

Parameter	Description	Definition
Apnea-hypopnea index (1/h)	The number of apnea and hypopnea events per hour	$\frac{n_{\mathrm{apnea}\kern0.17em \mathrm{events}}+{n}_{\mathrm{hypopnea}\kern0.17em \mathrm{events}}}{t_{\mathrm{analyzed}}}$
Oxygen desaturation index (1/h)	The number of desaturation events per hour	$\frac{n_{\mathrm{desaturation}\ \mathrm{events}}}{t_{\mathrm{analyzed}}}$
Apnea duration (s)	The duration of an apnea event	
Hypopnea duration (s)	The duration of a hypopnea event	
Desaturation duration (s)	The duration of a desaturation event	
Apnea proportion (%)	The proportion of apnea events out of all obstructive events	$\frac{n_{\mathrm{apnea}\kern0.17em \mathrm{events}}}{n_{\mathrm{apnea}\kern0.17em \mathrm{events}}+{n}_{\mathrm{hypopnea}\kern0.17em \mathrm{events}}}$

*n*_apnea events_, *n*_hypopnea events,_ and *n*_desaturation events_ denote the number of scored apnea, hypopnea, and desaturation events during the analyzed time. *t*_analyzed_ denotes the total analyzed time

**Fig. 1:**
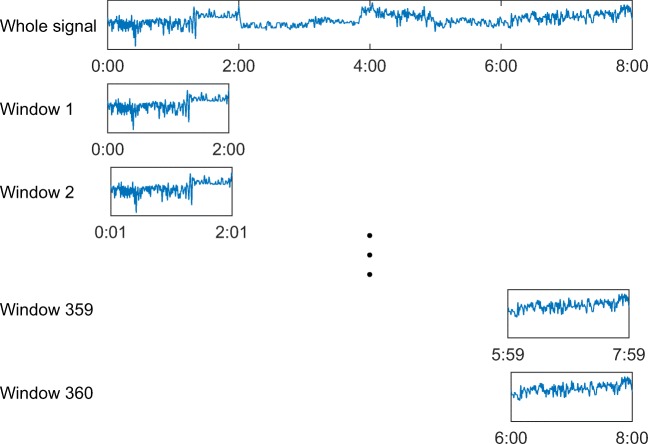
Illustration of the moving windows used for AHI_2h_ calculation. The 2-h window was moved from the beginning of the recording in 1 min interval to the end of the recording. AHI_2h_ was calculated for every window, and the window with the highest number of events was selected as the worst 2h

In the second part of the study, all 1989 patients were involved. The group thus included also subjects that did not fulfill the polygraphic criteria for diagnosis of OSA. In addition to full-night AHI, we calculated the number of apneas and hypopneas during a 2-h moving window for the whole night. Illustration of the moving window is presented in Fig. 1. The window with the highest number of events was selected as the worst 2 h. We will use notation AHI_2h_ for the number of apneas and hypopneas per hour during the worst 2 h. We will use AHI_standard_ for the standard full-night AHI.

Using AHI_2h_, all 1989 patients were re-classified to the standard OSA severity categories (non-OSA, mild, moderate, severe). The effect of re-classification on patients’ prognosis, when using AHI_2h_ instead of AHI_standard_ for severity classification, was investigated by comparing hazard ratios of the OSA severity categories. Cox proportional hazards model was used to calculate the hazard ratios for all-cause mortality, cardiovascular mortality, and non-cardiovascular mortality. OSA severity classification, age, body mass index (BMI), gender, smoking status, and the usage of continuous positive airway pressure (CPAP) ventilator were included in the model. In addition, Kaplan-Meier survival curves for each OSA severity category were calculated, with the classification based on AHI_standard_ and AHI_2h_.

The statistical significances of changes in the parameter values across the night were tested with linear one-way ANOVA contrasts test. Statistical significance of difference in patient demographic data were evaluated using Mann-Whitney *U* test and chi-square test. Statistical significances of the changes in the patients OSA severity classification when AHI_2h_ was used instead of AHI_standard_ were evaluated using chi-square test. All statistical tests were calculated with IBM SPSS statistics (version 23, IBM, Armonk, NY), and the limit for statistical significance was set to be *p* < 0.05. The hourly medians, hazard ratios, and Kaplan-Meier curves were calculated using MATLAB (2017b, MathWorks, Natick, MA, USA).

## Results

**Fig. 2:**
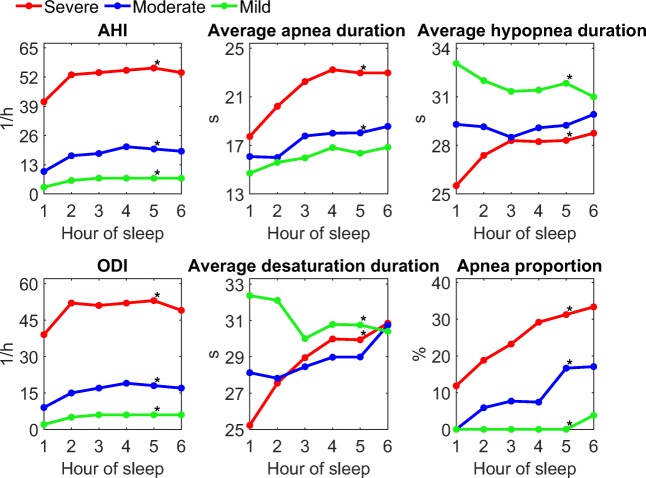
Hourly medians for apnea-hypopnea index (AHI), average apnea duration, average hypopnea duration, oxygen desaturation index (ODI), average desaturation duration, and apnea proportion of all obstructive events. An asterisk (*) denotes a statistically significant (*p* < 0.05) trend in the median based on linear one-way ANOVA contrasts test

In general, the hourly medians of parameters describing the severity of OSA increased towards morning (Fig. 2). Duration of apnea events increased significantly (*p* < 0.05) towards morning in moderate and severe OSA categories, while the duration of desaturation events increased significantly only in severe OSA category. Furthermore, AHI, ODI, and apnea proportion showed statistically significant increasing trends in all severity categories. The only exceptions to these trends were average hypopnea and desaturation event durations having statistically significant (*p* < 0.05) decreasing trends in mild OSA.

**Fig. 3:**
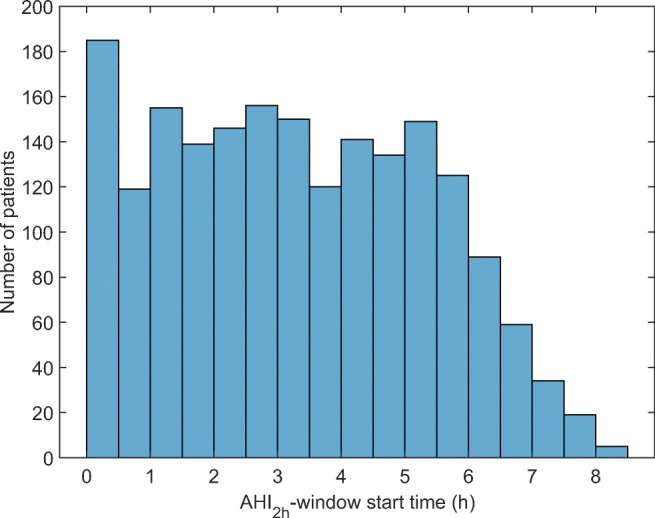
Histogram presenting the start times for the 2-h window with the highest AHI (AHI_2h_)

**Fig. 4:**
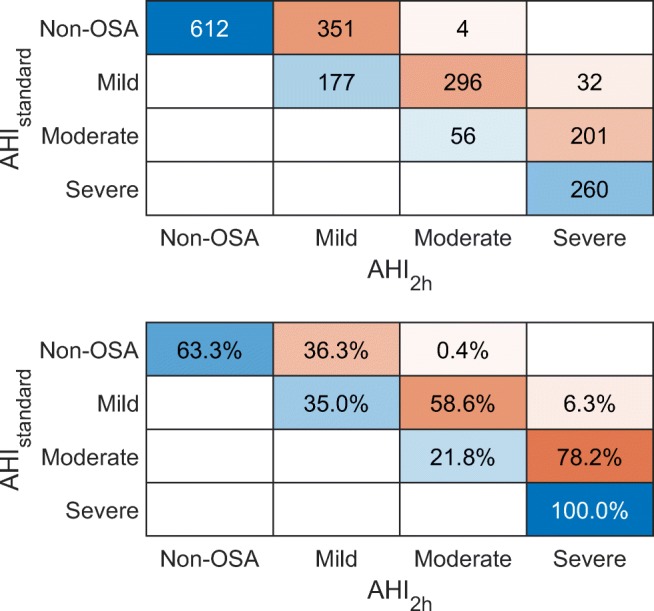
Absolute and normalized confusion matrices showing how the patients are classified to each obstructive sleep apnea category when the classification is based on full-night AHI (AHI_standard_) and the 2-h window with the highest AHI (AHI_2h_)

AHI_2h_ classified patients differently (*p* < 0.001, chi-square test) to OSA severity categories compared to AHI_standard_. The hours, which AHI_2h_ corresponds to, are presented as a histogram in Fig. 3. When AHI_2h_ was used, OSA severity classification changed to a more severe classification for a large portion of the patients (Fig. 4). This change was greatest in patients having moderate OSA based on AHI_standard_, as 78.2% of them received a classification of severe OSA when AHI_2h_ was used. By definition, AHI_2h_ is always at least as high as AHI_standard_ for every patient, and therefore, none of the patients were classified to have less severe OSA than what was observed based on AHI_standard_.

Using AHI_2h_ instead of AHI_standard_ had a significant effect on hazard ratios (Table 3). For all-cause, cardiovascular, and non-cardiovascular mortalities, AHI_2h_ led to greater hazard ratios in severe OSA than AHI_standard_. In addition, the hazard ratios consistently increased with increasing OSA severity classification when AHI_2h_ was used. This was not the case with AHI_standard_ as the hazard ratio of cardiovascular mortality was lower in mild OSA category than in non-OSA category (0.93 vs. 1), and the patients with moderate OSA had a higher hazard ratio than the patients with severe OSA (1.61 vs. 1.44). In addition, the hazard ratio of all-cause mortality was higher in moderate OSA category than in severe category (1.76 vs. 1.75) when AHI_standard_ was used.

**Table 3:** Hazard ratios (95% confidence interval) for all-cause mortality, cardiovascular mortality, and non-cardiovascular mortality in each OSA severity category when the severity classification was based on full-night AHI (AHI_standard_), and the 2 h with highest AHIs (AHI_2h_)

	OSA severity: mild	OSA severity: moderate	OSA severity: severe	Age (increase of 1 year)	BMI (increase of 1 unit)	Male gender	Smoking: yes	Smoking: quit	CPAP
All-cause mortality									
AHI_standard_	1.13 (0.84–1.51)	1.76* (1.27–2.44)	1.75* (1.22–2.51)	1.09* (1.08–1.10)	1.04* (1.02–1.05)	1.55* (1.15–2.08)	2.37* (1.82–3.09)	1.13 (0.86–1.49)	0.73* (0.55–0.96)
AHI_2h_	1.33 (0.93–1.91)	1.56* (1.07–2.27)	1.94* (1.33–2.83)	1.09* (1.07–1.10)	1.04* (1.02–1.05)	1.53* (1.14–2.06)	2.37* (1.82–3.09)	1.13 (0.86–1.48)	0.75* (0.57–0.98)
Cardiovascular mortality									
AHI_standard_	0.90 (0.57–1.44)	1.61 (0.98–2.63)	1.44 (0.83–2.51)	1.13* (1.10–1.15)	1.05* (1.03–1.08)	2.26* (1.38–3.70)	3.47* (2.29–5.23)	1.22 (0.79–1.88)	0.71 (0.46–1.09)
AHI_2h_	1.09 (0.62–1.92)	1.28 (0.71–2.30)	1.64 (0.92–2.90)	1.13* (1.11–1.15)	1.05* (1.03–1.08)	2.23* (1.36–3.65)	3.44* (2.28–5.19)	1.21 (0.79–1.87)	0.71 (0.47–1.08)
Non-cardiovascular mortality									
AHI_standard_	1.28 (0.88–1.87)	1.99* (1.28–3.11)	2.32* (1.46–3.70)	1.08* (1.06–1.10)	1.02* (1.00–1.05)	1.26 (0.86–1.82)	2.02* (1.43–2.84)	1.09 (0.77–1.54)	0.66* (0.46–0.96)
AHI_2h_	1.53 (0.96–2.45)	1.84* (1.13–3.01)	2.46* (1.49–4.07)	1.08* (1.06–1.10)	1.03* (1.00–1.05)	1.25 (0.86–1.81)	2.03* (1.43–2.86)	1.09 (0.77–1.54)	0.71 (0.49–1.02)

Hazard ratios were calculated with Cox proportional hazards model. Statistically significant (*p* < 0.05) hazard ratios are denoted with an asterisk (*)

**Fig. 5:**
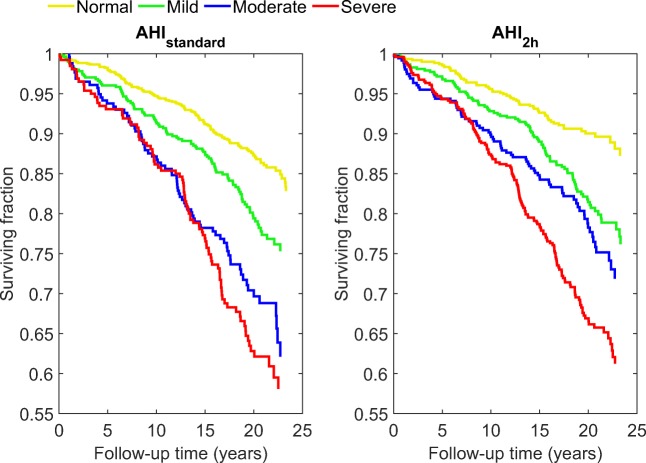
Kaplan-Meier survival curves for each obstructive sleep apnea category when the classification is based on full-night AHI (AHI_standard_) and the 2-h window with the highest AHI (AHI_2h_)

When OSA severity classification was based on AHI_2h_, the difference between the OSA severity categories in Kaplan-Meier survival curves was clear (Fig. 5). In contrast, when AHI_standard_ was used, the survival curves for moderate and severe OSA patients overlapped for the first 15 years of follow-up.

## Discussion

In accordance with our hypothesis, both the frequency and the severity of the obstructive events increased towards morning (Fig. 2). AHI and ODI increased the most between the first and the second hour and the difference between hours diminished after that. Average apnea duration and the proportion of apnea events increased relatively constantly towards morning. In moderate and severe OSA categories, average desaturation duration and average hypopnea duration also increased towards morning. This was not seen in the mild OSA category where the opposite trend was observed.

The implication of these findings to current practice of OSA diagnostics is that if the intra-night variation in OSA is not taken into account, the determination of OSA severity might not be optimal. Those patients whose AHI varies significantly on hour-to-hour basis might benefit from considering the intra-night variation in diagnostics. It is also worth noting that even though AHI increased towards morning, it increased less than average apnea duration and average desaturation duration indicating that the true severity of the disease cannot be fully assessed only based on the number or frequency on obstructive events.

AHI_2h_ was found to classify patients very differently compared to AHI_standard_ (Fig. 4). When using AHI_2h_ for classification, 36.3% of patients originally diagnosed to be non-OSA (i.e., AHI_standard_ < 5) were classified to have mild OSA. This means that they had at least 2 h of sleep with higher event frequency. In addition, four of the patients received a classification of moderate OSA, when AHI_2h_ was used. This implies that these patients spent most of the night without obstructive events but still had at least 2 h when the number of obstructive events was high. It is possible that this phenomenon could be explained by the fact that AHI is significantly elevated when sleeping in supine position [15]. The greatest change in OSA severity classifications however was seen with patients whose classification based on AHI_standard_ was moderate, as 78.2% of them received a classification of severe, when AHI_2h_ was used. This means that most patients diagnosed with moderate OSA have at least 2 h of sleep when the disease is severe.

The night-to-night variability of AHI_standard_ is known to be high, and AHI is also very dependent on sleeping position [16, 17]. This introduces a random element to the diagnostics of OSA. If a patient has positional OSA, the diagnosis might be mostly dependent on the amount of time spent on supine position during the recording. In addition, since EEG is not recorded in ambulatory polygraphy, it is not certain when exactly the patient is asleep. Periods of wake during the night naturally have a large effect on AHI since obstructive events only occur during sleep. Due to these reasons, it is possible that AHI_2h_ could be less susceptible to inter-night variability than AHI_standard_ and warrants to be investigated in the future.

Using AHI_2h_ for classification resulted in more consistent differences in hazard ratios between the OSA severity categories for all mortality types. In contrast, the classification based on AHI_standard_ resulted in hazard ratios that did not consistently increase with increasing OSA severity. There was also a relatively small difference between the non-OSA and mild categories when using AHI_standard,_ and the hazard ratio of cardiovascular mortality was even lower in the mild OSA category than in the non-OSA category. In addition, the hazard ratios of all-cause and cardiovascular mortalities were higher in the moderate OSA categories than in the severe categories (1.76 vs. 1.75 and 1.61 vs. 1.44). According to these observations, AHI_standard_ may not be optimal for differentiating between non-OSA and mild OSA and between moderate and severe OSA. This again implies that patients might not be optimally diagnosed when AHI_standard_ is used.

We observed that the hazard ratios of the severe category when using AHI_2h_ were slightly higher than in the case of AHI_standard_. This means that using AHI_2h_, the difference in relative mortality risks between patients receiving non-OSA and severe classification is greater. This further supports the idea that AHI_standard_ does not differentiate the patients with highest risk for mortality in sufficient manner, but these patients can be better captured using AHI_2h_. The relative mortality risks between AHI_standard_ and AHI_2h_ are not known however, because different group of patients are classified as non-OSA.

It is interesting to note that the hazard for all-cause and cardiovascular mortalities caused by smoking was higher than the hazard caused by any severity of sleep apnea. Increase of age and BMI also increased the hazard for all types of mortalities. In addition, male gender also increased the hazard for each mortality type but especially for cardiovascular mortality where the hazard for men was more than twice as high as for women. Additionally, the use of CPAP clearly reduced the hazard for all types of mortalities.

By inspecting the survival curves in Fig. 5, similar observations, as with hazard ratios, can be made. AHI_2h_ is better at differentiating between the severity categories. We observed that in non-OSA, mild, and moderate categories, the average survival time is longer when the classification is based on AHI_2h_. This again could mean that when AHI_standard_ is used for classification, OSA severity of some patients, especially those belonging in non-OSA and moderate OSA categories, is underestimated. Although AHI_2h_ seems to show clearer agreement with estimated mortality risks than the standard AHI, it does not take into account the duration of the obstructive event or the severity of the desaturation caused by the event. Therefore, additional or even more comprehensive parameters are needed to completely assess all aspects of OSA severity.

### Limitations

Although a large pool of patients with long follow-up and thorough analysis of recorded signals was investigated, this study is not without limitations. The main limitation in the first part of the study was that only the first 6 h of sleep were analyzed. This decision was made because almost all patients (1840 of 1989) had at least 6 h of recording time and using longer analysis window would have significantly reduced the pool of patients. However, some of the patients had much longer recording time than 6 h and it is possible that somewhat different changes in obstructive event severity could have been observed past the first 6 h. However, to study this reliably, a much larger sample size would have been needed since only 27% of the patients included in this study had recording time of 8 h or more.

In addition, a limitation in the ambulatory recordings is that they did not include EEG recording. For this reason, hypopneas associated only with arousals could not be detected and are thus not included in the data. In addition, the exact sleep time cannot be detected without EEG. It is therefore possible that the lower amount of obstructive events occurring in the beginning of the night is at least partially caused by lower sleep efficiency in that part of the night. However, ambulatory polygraphy without EEG registration is widely used and accepted as an alternative for polysomnography [18]. Therefore, same issues are also present in standard OSA diagnostics. Thus, we consider that this does not undermine the present finding that AHI_standard_ may not be the most optimal parameter for diagnosis. Additionally, the polygraphic recordings were scored by five different scorers whose scoring agreement has not been evaluated.

A limitation in the proportional hazards model was that additional background morbidities such as coronary artery disease, hypertension, diabetes, or alcohol use could not be included in the model since this data was only available to a very limited number of patients. In addition, although the AHI_2h_ seems to be a better predictor of the mortality risk, the confidence intervals are too wide for reaching statistical significance. Another limitation in the Cox proportional hazards model is that the patients classified as non-OSA are not actually healthy but instead suspected OSA patients typically having other background morbidities. All patients were referred to polygraphy due to suspected OSA, and the non-OSA patients in this study are those that were simply not diagnosed with OSA. It is likely that if truly healthy controls would have been included, it would have increased the relative mortality risk of patients with OSA.

## Conclusions

To conclude, the main finding of the study is that the severity of individual obstructive events varies significantly hour-by-hour during a single night and the obstructive events that occur at the beginning of the night are less severe and less frequent compared to the events occurring later during the night. In addition, when patients are classified based on the standard AHI, the OSA severity classification does not optimally reflect the mortality risks. Therefore, the widely clinically used standard full-night AHI might not be the best parameter for predicting the mortality risk related to OSA and thus may not be the best parameter for classification of OSA severity.
